# Thyrotoxic Periodic Paralysis: A Unique Case Highlighting the Diagnostic Challenges and Management

**DOI:** 10.7759/cureus.73275

**Published:** 2024-11-08

**Authors:** Jawad Atrash, Tariq Musleh, Yazan Naji, Vida Said, Monjed Saraheen

**Affiliations:** 1 Internal Medicine Department, Saint Joseph Hospital, Jerusalem, PSE

**Keywords:** acute flaccid paralysis, beta-blockers, hypokalemia, potassium replacement, thyrotoxicosis

## Abstract

Thyrotoxic periodic paralysis (TPP) is a rare complication primarily associated with thyrotoxicosis, particularly in individuals with Graves’ disease. While more common in males aged 20 to 40, it can occur across all ethnic backgrounds. It is the most common type of acquired periodic paralysis. The condition is triggered by factors like exercise, stress, diet, and certain medications. The diagnosis is confirmed by severe hypokalemia and elevated thyroid hormones in the presence of acute flaccid paralysis. Immediate treatment involves correcting hypokalemia, while long-term management focuses on normalizing thyroid levels. In this article, we will report a case of a 30-year-old Middle Eastern man who presented to the emergency room with severe muscle weakness following extensive exertion and a high-carbohydrate diet. Physical examination revealed motor weakness in all limbs, particularly his lower limbs. Initial tests showed marked hypokalemia and biochemical thyrotoxicosis with suppressed thyroid-stimulating hormone (TSH) and elevated free triiodothyronine (T3) and free thyroxine (T4). An electrocardiogram (ECG) indicated signs of severe hypokalemia with an atrioventricular (AV) block. After he was diagnosed with TPP, the patient received oral and intravenous potassium infusions and was started on anti-thyroid medications, most importantly beta (β)-blockers. Following acute treatment, his potassium levels normalized, and he regained full muscle function. Ultimately, he was maintained on anti-thyroid medications at discharge to maintain an euthyroid state in order to prevent future recurrences of symptoms. Consequently, in patients presenting with acute flaccid paralysis, potassium level, and thyroid function should be investigated in order to promptly diagnose complications of thyrotoxicosis and to start an early appropriate combined therapy. The early and rapid management of TPP can prevent serious cardiopulmonary complications.

## Introduction

Thyrotoxic periodic paralysis (TPP) is a very rare complication of thyrotoxicosis in the White population but is more frequently reported in individuals of Asian descent [1]. However, in recent decades, an increasing number of patients from all racial and ethnic backgrounds have been reported [2]. It is most commonly a complication of Graves' disease [2]. Hypokalemia, with associated flaccid paralysis, and signs of hyperthyroidism are the hallmark [1]. Despite the higher incidence of hyperthyroidism in females, more than 95% of TPP occurs in males [3] and typically occurs between the ages of 20 and 40 years [1]. Hypokalemia and muscle paralysis result from a sudden intracellular shift of potassium and are not due to potassium deficiency. Increased sodium-potassium ATPase pump activity due to excess thyroid hormone levels and enhanced insulin response in patients with TPP are postulated to contribute to severe hypokalemia. Clinical features of hyperthyroidism in patients with TPP may be subtle [4]. It is precipitated by strenuous exercise, a high-carbohydrate diet, stress, infection, alcohol, albuterol, and corticosteroid therapy [5]. The proximal muscles are more severely affected than the distal ones. The lower limbs are usually affected first, followed by the girdle muscles and the upper limbs. The sensory system remains unaffected [4]. The presence of both severe hypokalemia and biochemical thyrotoxicosis (increased serum levels of triiodothyronine (T3) and thyroxine (T4) with suppressed thyroid-stimulating hormone (TSH)) in the presence of bilateral symmetrical flaccid paralysis confirms the diagnosis of TPP. Correcting hypokalemia is mandatory for immediate reversal of paralysis. However, preventing future TPP attacks is achieved by restoring normal thyroid status [6].

Here, we report a case of a 30-year-old Middle Eastern man with free past medical and surgical histories who presented to the emergency department in Saint Joseph Hospital in Jerusalem with acute symmetrical flaccid paralysis of his lower limbs with marked electrocardiogram (ECG) changes related to severe hypokalemia.

## Case presentation

A 30-year-old Middle Eastern man, from Jerusalem, married, with free past medical and surgical histories, presented to Saint Joseph Hospital’s emergency room in Jerusalem with severe generalized muscle weakness more pronounced in his lower extremities and an inability to stand up or walk, associated with progressive muscle cramps of two days duration. The weakness started in his lower limbs and then gradually progressed to the upper limbs over a period of two days. He also reported intermittent palpitations. He had no similar episodes before. The patient mentioned extensive exertion as he was at a wedding party and mentioned eating a lot of sweets and carbohydrates in the last week. He denied the use of diuretics, laxatives, alcohol, recreational drugs, or any new medications. There was no history of vomiting, diarrhea, dysphagia, fever, chills, shortness of breath, chest pain, joint pain, skin rash, headache, dizziness, slurred speech, or loss of consciousness. There was no family history of autoimmune diseases or any relative neurological disorders.

On physical examination, the patient looked ill, weak, conscious, alert, oriented to time, place, and person, and not in respiratory distress. His blood pressure was 140/64 mmHg, his heart rate was 95 beats per minute, his temperature was 36.8°C, his respiratory rate was around 20 breaths per minute, and his oxygen saturation (Sp0_2_) was 97% on room air. He had a mildly non-tender, enlarged thyroid with a firm consistency. No thyroid bruits were auscultated. He had no cyanosis, jaundice, conjunctival hemorrhage, photophobia, lymphadenopathy, or exophthalmos. An irregular heart rate with no audible murmurs. The chest and abdominal examinations were unremarkable. Neurological examination showed decreased muscle strength and tendon reflexes in all four limbs (the power was 1/5 in both lower limbs and 3/5 in both upper limbs according to the Medical Research Council (MRC) muscle power scale). His cranial nerves were intact with no sensory deficits. There was no bladder or bowel dysfunction. No lower limb edema was identified.

**Figure 1:**
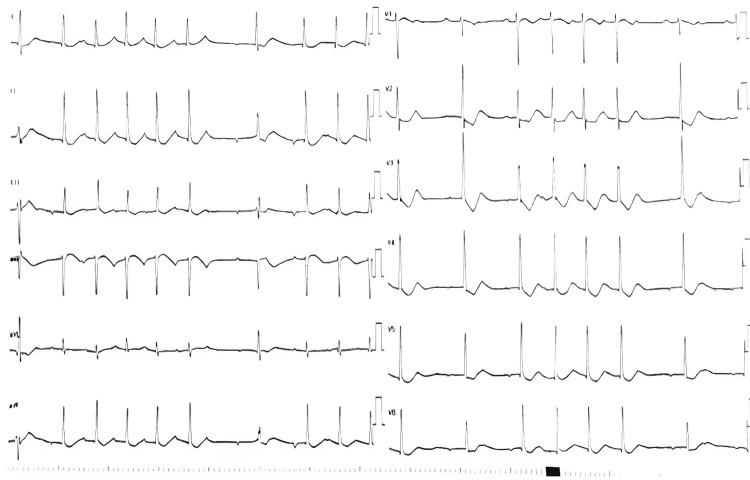
The patient's ECG at presentation Signs of severe hypokalemia: sinus rhythm, premature ventricular contractions (PVCs), premature atrial contractions (PACs), U wave at V4 through V6 leads, prolonged PR interval, diffuse ST depression, shallow T waves, and AV block; heart rate: 68 beats per minute were noted.

In the emergency department, his initial laboratory investigations showed marked hypokalemia of 1.5 mEq/L (reference range, 3.5-4.9 mEq/L) with normal acid-base status (pH) and spot urinary potassium of 39 mEq/L (reference range, 20-80 mEq/L). His serum creatine phosphokinase (CPK) level was 1336 (reference range, 20-190 IU/L), his serum phosphorus level was 1.11 mg/dL (reference range, 2.5-4.5 mg/dL), and his serum magnesium level was 1.63 mg/dL (reference range, 1.6-2.6 mg/dL). Calcium, albumin, and kidney function tests were all within the normal range. Other lab results included a white blood cell count of 9.15 billion cells/L, hemoglobin level of 15.7 g/dL, and platelet count of 200 billion cells/L with an otherwise normal differential. Urinalysis was normal, and C-reactive protein was 2 mg/dl (reference range, 0-5). The results of the labs at presentation are detailed in Table 1. Initial ECG showed sinus rhythm, premature ventricular contractions (PVCs), premature atrial contractions (PACs), U wave at V4 through V6 leads, prolonged PR interval, diffuse ST depression, shallow T waves, and AV block, heart rate: 68 beats per minute (Figure 1). An initial diagnosis of hypokalemic periodic paralysis was made. The patient was admitted for further work up to the intensive care units (ICU) under the care of the internal medicine team. He was started on an intravenous infusion of potassium chloride (10 mEq/hour) via a peripheral intravenous line, and he was also placed on an oral potassium replacement (600 mg prolonged-release capsules three times a day) according to protocol.

**Table 1:** The patient's laboratory investigations at presentation

Test	Patient’s result	Reference range
Creatinine	0.58 mg/dL	0.7­-1.2 mg/dL
Sodium	139 mEq/L	135-149 mEq/L
Potassium	1.5 mEq/L	3.5-5.9 mEq/L
Chloride	103 mEq/L	90-110 mEq/L
Phosphorus	1.11 mg/dL	2.5-4.5 mg/dL
Magnesium	1.63 mg/dL	1.6-2.6 mg/dL
Calcium	9.41 mg/dL	8.6-10 mg/dL
Albumin	3.9 g/dL	3.2-4.94 g/dL
Random blood sugar	111 mg/dL	75-110 mg/dL
Creatine phosphokinase (CPK)	1336 IU/L	20-190 IU/L
C-reactive protein (CRP)	2 mg/dL	0-5 mg/dL
Bicarbonate (HCo3)	22 mmHg	24-28 mmHg
Hemoglobin	15.7 g/dL	12.6-17.4 g/dL
White blood cell count	9.15 billion cells/L	4.5-11 billion cells/L
Platelets count	200,000 billion cells/L	150-440 billion cells/L
Spot urinary potassium	39 mEq/L	20-80 mEq/L

**Figure 2:**
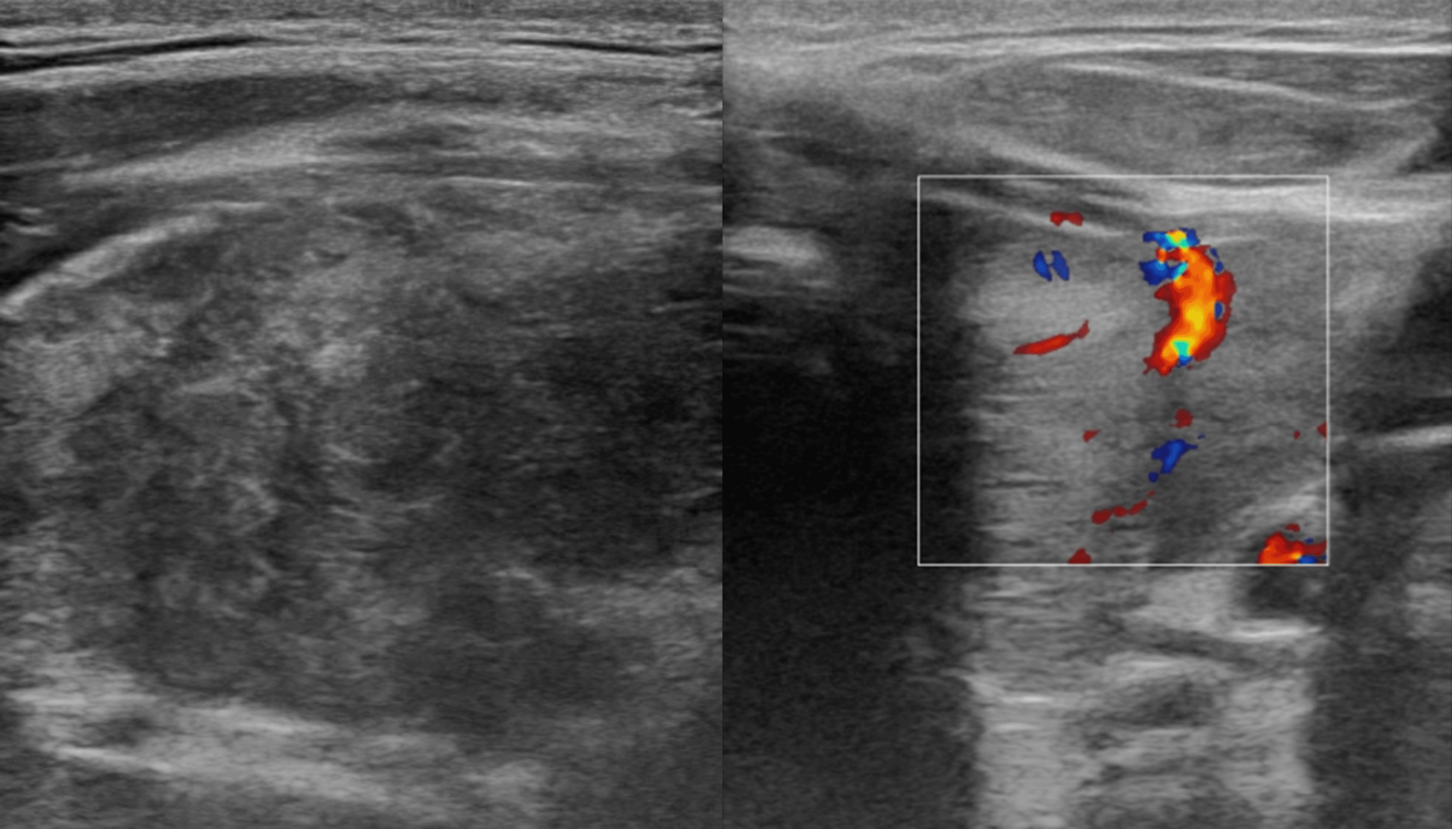
Thyroid ultrasound images Thyroid gland with heterogenous echotexture, geographic diffuse hypoechoic areas, and mildly increased parenchymal vascularity on color Doppler

To determine the cause of his hypokalemic paralysis, we checked his thyroid function tests, which showed biochemical thyrotoxicosis: fully suppressed TSH of 0.006 mU/L (reference range, 0.27-4.2 mU/mL) with elevated free T3 of 21.06 (reference range, 3.1-6.8 pmol/L) and elevated free T4 of 47.6 pmol/L (reference range, 12-22 pmol/L) (Table 2). His anti-thyroid peroxidase (TPO) antibodies and TSH-receptor antibodies were not checked due to the unavailability of their kit in the hospital's laboratory department. A thyroid ultrasound revealed that both thyroid lobes are enlarged with diffuse heterogeneous hypoechoic texture and mildly increased parenchymal vascularity on color Doppler (Figure 2).

**Table 2:** The patient's thyroid function test at presentation indicating signs of severe hyperthyroidism.

Test	Patient’s result	Reference range
Thyroid-stimulating hormone (TSH)	0.006 mU/mL	0.27–4.2 mU/mL
Free thyroxine (T4)	47.6 pmol/L	12–22 pmol/L
Free triiodothyronine (T3)	21.06 pmol/L	3.1–6.8 pmol/L

**Figure 3:**
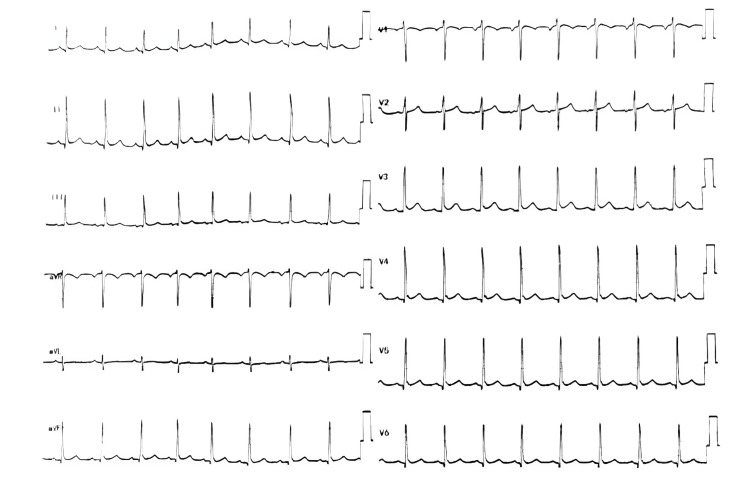
The patient's ECG post treatment indicating a normal pattern with resolution of all the previous ECG abnormalities that were related to severe hypokalemia after normalization of serum potassium level.

Thyrotoxic periodic paralysis was diagnosed. The patient was given IV propranolol 1 mg as a non-selective beta (β)-blocker. Accordingly, the endocrinology team recommended initiating anti-thyroid medications: methimazole 20 mg orally two times a day and propranolol 20 mg orally three times a day. With adequate potassium replacement and follow-up of its level (serum potassium levels reached 5.88 mEq/L) over a period of 12 hours, the patient showed progressive and rapid complete resolution of all of his symptoms, regaining full muscle power in all extremities and his ability to walk after normalization of serum potassium level. Laboratory results post treatment are detailed in Table 3. The ECG was repeated after normalization of potassium level on the next day of admission, and it returned to regular sinus rhythm with no other abnormality (Figure 3). Serial measurements of his serum potassium level in the hospital remained within normal limits without the need for oral potassium supplementation.

**Table 3:** The patient's laboratory investigations post treatment

Test	Patient’s result	Reference range
Potassium	4.36 mEq/L	3.5–5.9 mEq/L
Sodium	137 mEq/L	135-149 mEq/L
Chloride	100 mEq/L	90-110 mEq/L
Creatine phosphokinase (CPK)	166 IU/L	20-190 IU/L

According to the above-mentioned rapid response to management with complete resolution of symptoms after normalization of hypokalemia and controlling of thyrotoxicosis, TTP was confirmed. Serum potassium level on discharge was 4.36 mEq/L and CPK level was 166 IU/L. The patient was advised to stick to his prescribed anti-thyroid medications with regular follow-ups with an endocrinologist and regular repetition of thyroid profile every six weeks to monitor for treatment effectiveness. In addition, the patient was informed about the common potential adverse effects of therapy, like agranulocytosis in methimazole therapy, and the importance of monitoring and recognizing it. The patient was discharged home with the diagnosis of TPP secondary to thyrotoxicosis, which was exacerbated by heavy exertion and a high-carbohydrate meal. He was advised to avoid the previously mentioned exacerbating factors to prevent future relapsing of symptoms.

## Discussion

Hypokalemic periodic paralysis is a rare condition that can be classified as either primary (familial) or secondary (environmental) [5]. Secondary causes include hyperthyroidism and various disorders such as hyperaldosteronism, diabetic ketoacidosis, nephrotic syndrome, certain medications, acute tubular necrosis, and conditions leading to diarrhea or vomiting [5]. Thyrotoxic periodic paralysis is particularly prevalent among Asian men, including those of Chinese, Japanese, Vietnamese, Filipino, and Korean descent, with Graves’ disease being the most frequent cause of hyperthyroidism linked to TPP [5]. While thyrotoxicosis typically affects women, TPP is predominantly observed in males aged in their twenties to forties [7].

Due to the rarity of TPP, it’s important to consider other diagnoses like familial hypokalemic periodic paralysis, myasthenia gravis, Guillain-Barré syndrome, viral and inflammatory myopathies, transverse myelitis, cord compression, and other electrolyte imbalances [5]. These conditions can usually be excluded through patient history, physical examination, and further testing [5]. Many individuals with TPP do not show obvious symptoms or signs of thyrotoxicosis [5]. Factors that may trigger TPP in patients with existing thyrotoxicosis include high-carbohydrate and high-salt diets, alcohol intake, trauma, menstrual cycles, infections (such as viral gastroenteritis), certain medications (like steroids, diuretics, epinephrine, acetazolamide, and insulin), and vigorous exercise [8]. These events often coincide with increased release of epinephrine or insulin, which drives potassium into cells and results in low blood potassium levels [8].

Patients typically present with sudden onset of symmetrical proximal weakness in the lower limbs, occurring in the early morning or after resting following intense physical activity and/or a high-carbohydrate meal [7]. Hyporeflexia or areflexia are common [8]. Acute episodes may be preceded by muscle aches, cramps, and stiffness [7]. Though paralysis rarely affects ocular, bulbar, or respiratory muscles, respiratory impairment has been noted [7]. Sensation and consciousness are usually unaffected [7]. Between episodes, neurological examinations are typically normal [8]. Signs of thyrotoxicosis, such as warm, moist skin, fever, tachycardia, exophthalmos, or goiter, may be subtle but are critical for diagnosis [7]. Serious complications can include dysrhythmias, respiratory failure, and death [7].

In our case, the patient experienced severe proximal muscle weakness, starting in the lower limbs and progressing to the upper limbs over two days. He had a power rating of approximately 1/5 in his lower limbs and 3/5 in his upper limbs and showed no bladder or bowel dysfunction. The weakness followed extensive physical exertion at a wedding party, during which he consumed a significant amount of sweets and carbohydrates in the week leading up to his ER visit. A thyroid function test confirmed the diagnosis of TPP, revealing biochemical thyrotoxicosis alongside severe hypokalemia and flaccid paralysis. Although cardiac arrhythmias like tachycardia, atrial fibrillation, paroxysmal supraventricular tachycardia, or ventricular fibrillation are rare, they have been documented during episodes [9]. The ECG may display hypokalemia-related changes, such as ST segment depression, reduced T wave amplitude, and increased U wave amplitude [9], as seen in our patient's ECG upon presentation. The paralysis can last anywhere from three to 96 hours, resolving in the reverse order of onset [7].

The aim of therapy is to quickly replenish potassium while lowering thyroid hormone levels with careful monitoring for rebound hyperkalemia during recovery [10], as rapid cellular shifts in potassium during therapy can cause severe adverse complications. Intravenous potassium is essential for patients with severe symptoms or arrhythmias [10]. This method provides a quicker response compared to oral supplementation [10]. Additionally, non-selective β-blockers can alleviate neuromuscular symptoms by minimizing the intracellular movement of potassium and addressing signs of thyrotoxicosis [11]. The primary objective is to lower thyroid hormone levels and achieve an euthyroid state to prevent future episodes of TPP while monitoring for potential adverse effects of therapy [12]. Depending on the cause of hyperthyroidism, treatments such as anti-thyroid medications, radioactive iodine, or surgery may be required [12]. It’s also important to avoid triggering factors like intense exercise and high-carbohydrate meals [12].

## Conclusions

Thyrotoxic periodic paralysis represents an acquired form of hypokalemic periodic paralysis. Thyrotoxic periodic paralysis is secondary to thyrotoxicosis, and it usually mimics the clinical presentation of other forms of familial hypokalemic periodic paralysis. Any cause of hyperthyroidism can be associated with TPP, most commonly in Graves’ disease. The diagnosis of TPP is made when a patient presents with lower extremities paralysis that is associated with hypokalemia and hyperthyroidism. It is necessary that an early diagnosis of TPP is made to administer definitive treatment and prevent morbidity and mortality, mainly cardiopulmonary complications. Treatment with potassium supplementation and non-selective β-blockers should be started upon diagnosis, and the serum potassium level should be monitored frequently to prevent rebound hyperkalemia, as rapid cellular shifts in potassium during therapy can cause severe adverse complications. Resolution of symptoms will be rapid and complete following normalization of serum potassium levels. Long-term management includes regular follow-up, avoiding exacerbating factors, monitoring for potential adverse effects of therapy, and addressing the underlying cause of hyperthyroidism with definitive therapy to prevent future recurrence of symptoms. Early detection, effective treatment, and prevention of rebound hyperkalemia became recognized after an increased awareness of this disorder was achieved.

## References

[1] Erem C (2005). Thyrotoxic hypokalemic periodic paralysis in a Turkish male with Graves' disease: a rare case report and review of the literature. Endocrine.

[2] Pothiwala P, Levine SN (2010). Analytic review: thyrotoxic periodic paralysis: a review. J Intensive Care Med.

[3] Hsieh MJ, Lyu RK, Chang WN (2008). Hypokalemic thyrotoxic periodic paralysis: clinical characteristics and predictors of recurrent paralytic attacks. Eur J Neurol.

[4] Kung AW (2006). Clinical review: thyrotoxic periodic paralysis: a diagnostic challenge. J Clin Endocrinol Metab.

[5] Mohamed HN, Ghedi AKA, Ozturk S, Jeele MOO, Bashir AM (2023). Hypokalemic periodic paralysis as the first sign of thyrotoxicosis- a rare case report from Somalia [PREPRINT]. Res Sq.

[6] Khadka S, K C I, Rayamajhi RJ, Dawadi P, Budhathoki P (2019). Thyrotoxic periodic paralysis with hypokalemia in an adult male from Nepal: a case report. JNMA J Nepal Med Assoc.

[7] Lam L, Nair RJ, Tingle L (2006). Thyrotoxic periodic paralysis. Proc (Bayl Univ Med Cent).

[8] Fontaine B, Lapie P, Plassart E, Tabti N, Nicole S, Reboul J, Rime-Davoine CS (1996). Periodic paralysis and voltage-gated ion channels. Kidney Int.

[9] Ober KP (1992). Thyrotoxic periodic paralysis in the United States. Report of 7 cases and review of the literature. Medicine (Baltimore).

[10] Venance SL, Cannon SC, Fialho D (2006). The primary periodic paralyses: diagnosis, pathogenesis and treatment. Brain.

[11] Yu TS, Tseng CF, Chuang YY, Yeung LK, Lu KC (2007). Potassium chloride supplementation alone may not improve hypokalemia in thyrotoxic hypokalemic periodic paralysis. J Emerg Med.

[12] Tessier JJ, Neu SK, Horning KK (2010). Thyrotoxic periodic paralysis (TPP) in a 28-year-old Sudanese man started on prednisone. J Am Board Fam Med.

